# Mindful awareness as a mechanism of change for natural childbirth in pregnant women with high fear of childbirth: a randomised controlled trial

**DOI:** 10.1186/s12884-022-04380-0

**Published:** 2022-01-19

**Authors:** Irena K. Veringa-Skiba, Kelly Ziemer, Esther I. de Bruin, Ed J. de Bruin, Susan M. Bögels

**Affiliations:** 1 Research Institute of Child Development and Education (RICDE), 8 Faculty of Social and Behavioural Sciences, University of Amsterdam, Research Priority Area Yield, Nieuwe Achtergracht 127, 1018 WS Amsterdam, The Netherlands; 2 University of California-Berkeley, School of Social Welfare, Haviland Hall, Berkeley, CA 94709 USA; 3 UvA-minds, Academic Treatment Center of the University of Amsterdam, Banstraat 29, 1071 JW Amsterdam, The Netherlands; 4 Department of Psychology, Health & Technology, University of Twente, PO Box 217, 7500 AE Enschede, The Netherlands

**Keywords:** Fear of childbirth, Mindfulness, MBCP, Natural childbirth, Obstetric interventions

## Abstract

**Background:**

Mindfulness-Based Childbirth and Parenting (MBCP) is effective in increasing natural childbirth in pregnant women with high fear of childbirth (FOC) as compared to enhanced care as usual (ECAU). We aimed to examine through which pathway of action MBCP reaches this effect, based on a model of approaching or avoiding the challenges related to childbirth.

**Methods:**

One hundred eleven pregnant women with high FOC were measured pre- and post-intervention on FOC (emotion pathway), catastrophic beliefs about labour pain (cognition pathway) and mindful awareness (attention pathway). A multiple mediation model was used to examine through which pathway the mechanism of change operated in relation to approach (i.e., natural childbirth) versus avoidance (i.e., self-requested caesarean section).

**Results:**

It was found that greater mindful awareness (18% R^2^ = 0.18, *F*[1107] = 22.77, *p* < 0.0001) was the only significant mechanism of change operating through the attentional pathway leading to natural childbirth. More specifically, nonreactivity to inner experience (a facet of mindful awareness) showed to be the strongest mechanism of change. More extensive meditation practice was positively associated with natural childbirth; however, the number of completed MBCP sessions was not associated with the outcome.

**Conclusions:**

An increase in mindful awareness was the strongest mechanism of change for better adaptation to the challenges of childbirth. Decreases in neither FOC nor catastrophic beliefs about labour pain were identified as mechanisms of change. Additionally, the more one meditated, the more one was inclined towards a natural childbirth. MBCP enhances adaptation to the challenges of childbirth and less use of obstetric interventions in the presence of high FOC.

**Trial registration:**

The Netherlands Trial Register (NTR; 4302).

**Supplementary Information:**

The online version contains supplementary material available at 10.1186/s12884-022-04380-0.

## Background

Mindfulness-based programs (MBPs) have shown to be effective for a variety of psychological and physical conditions including depression, anxiety, stress and chronic pain in clinical and non-clinical populations [1–3]. MBPs also showed potential in reducing anxiety, depression and stress in pregnant women as demonstrated with pooled results of uncontrolled studies [4]. However, pooled results of controlled studies did not show the same outcomes [4]. Promising are novel findings from two randomized controlled trials (RCTs) from Sweden and The Netherlands on the effects of the Mindfulness-Based Childbirth and Parenting (MBCP) programs in pregnant women on perceived stress and symptoms of depression [5], and high levels of fear of childbirth (FOC) [6]. The Swedish RCT showed that MBCP is more effective in decreasing perceived stress and being at risk for perinatal depression as compared to a Lamaze childbirth course [5]. The Dutch RCT focused on pregnant women with high FOC and contributed to the research by measuring, next to fear of childbirth, the actual childbirth process as outcome variable. Approach versus avoidance of childbirth was assessed, with better adaptation expressed as natural childbirth versus maladaptation expressed as a childbirth with (unneeded) obstetric interventions. MBCP, in comparison to enhanced care as usual (ECAU), was found to have a medium positive effect on the reduction of FOC and catastrophic beliefs about labour pain; a large effect on the reduction in willingness to undergo obstetric interventions in the absence of obstetric indications; a reduction in received self-requested caesarean section (sCS) and epidural analgesia (EA); and remarkably, women after MBCP were two times more likely to undergo natural birth (spontaneous vaginal birth without obstetric interventions) [7]. Yet, it is unknown *how* MBCP has impacted an increase in natural childbirths in the presence of high levels of FOC.

To answer this question, we have presented a theoretical model of two opposite behavioural responses to the challenges of childbirth, namely avoiding versus approaching [8]. This model was derived from cognitive theory [9] on fear and anxiety, which emphasizes the interrelationship between negative emotions (anxiety/fear), biased cognitions (catastrophic beliefs), biased attention (threat-focused), and maladaptive behaviours (avoiding) [10]. Avoiding is a maladaptive behaviour since it becomes more harmful than helpful in dealing with the challenges of childbirth, such as undergoing a sCS. The proposed model has been robustly supported with empirical findings that pregnant women, who plan to avoid the challenges of a natural childbirth by requesting and undergoing obstetric interventions such as sCS or EA, experienced high FOC [11–15]; had catastrophic beliefs about labour pain [16, 17]; and appraised childbirth as threatening and focused attention on potential threatening aspects of childbirth [17, 18]. Further, high FOC is also associated with poorer adaptation to childbirth, thus a longer duration of dilatation period during labour [19], and even the use of an emergency CS [20]. On the other hand, balanced emotions, realistic beliefs and unbiased attention (mindful awareness) would lead to more adaptive behaviour such as approaching the challenges of childbirth, by undergoing a natural childbirth. It can be concluded that high FOC is strongly related to obstetric interventions during childbirth due to pregnant women’s requests or problems occurring during birth.

In our model, three possible pathways of action leading to avoiding or approaching the challenges of childbirth in pregnant women with high FOC were hypothesized as: (I) an emotion pathway – a change in FOC, (II) a cognition pathway – a change in catastrophic beliefs about labour pain, and (III) an attention pathway – a change in mindful awareness. Particularly, the change in mindful awareness, was hypothesised to be an important mechanism of change by which specifically MBCP would help in approaching rather than avoiding the challenges of childbirth [18].

The attention pathway in this study is defined as mindful awareness of the moment-to-moment experience, and it can be cultivated during mindfulness meditations [21]. Mindfulness meditation is at the core of MBPs, and mindful awareness is typically described as “a form of non-judgmental, nonreactive attention to experiences occurring in the present moment, including cognitions, emotions, and bodily sensations as well as sights, sounds, smells, and other environmental stimuli” [22]. During mindfulness meditations, participants observe a variety of experiences that may arise, while cultivating an attitude of open interest to these experiences. This allows the experiences to exist, despite a residual willingness or reactivity to change or a desire to escape from them, even if they feel unpleasant. Mindfulness meditation helps the practitioner to realize that physical sensations, thoughts and emotions are continuously changing, as they are arising and disappearing in the awareness. Mindfulness meditations are born from Eastern meditation traditions, which emphasize that the practice of mindfulness leads to less suffering and more wisdom, compassion and equanimity [23].

Traditional mindfulness meditations have been successfully adapted for use in Western mental health approaches. For instance, Mindfulness-Based Stress Reduction (MBSR) [24] and Mindfulness-Based Cognitive Therapy (MBCT) [25] have become widely used in health care settings and with both clinical and non-clinical populations to reduce human suffering caused by psychological and physical vulnerabilities. Extensive research on the mechanisms of change, in which MBSR and MBCT influenced negative experiences in people with physical and/or psychological conditions, suggested mindful awareness as a universal mechanism of positive change [1]. Behavioural self-regulation and increased behaviour adaptation under stressful circumstances are examples of positive change with mindful awareness [26, 27]. Other research on mindful awareness also found that a greater mindful awareness acted as a mechanism of change for MBSR in non-pregnant populations with anxiety disorders in reducing worries [28], anxiety, and avoidance symptoms [29]. One of the instruments to measure mindful awareness is the five facets of mindfulness as defined by Baer and colleagues [30].

## Methods

### Aims

In the present study, we evaluated three possible pathways of action of avoiding (e.g., having a sCS) versus approaching (e.g., having a natural childbirth) the challenges of childbirth in pregnant women with high FOC. We hypothesized that following MBCP, several mechanisms of change would contribute to natural childbirth: the change from high to lower FOC (emotional pathway), the change from high to lower catastrophic beliefs about labour pain (cognitive pathway), and the change from low to higher mindful awareness (attention pathway). Additionally, we tested whether the number of completed MBCP sessions and the minutes of meditation practice at home were associated with the outcome.

### Procedure and subjects

In this study, we analysed the “I’ve Changed My Mind” RCT-data in which 141 pregnant women without a priori (medical) restrictions for natural childbirth experiencing high FOC were randomized to MBCP (*n* = 75) or ECAU (*n* = 66) [6]. There were no significant pre-intervention differences between conditions for demographic predictors and outcome measures [6]. Below, a summary of the methodological details most pertinent to the current study are presented. The study’s procedure, which includes the rates of recruitment, reasons for refusal, exclusion, withdrawal and attritions, as well as the course of randomization and masking, has been published previously [6]. Inclusion criteria were an age ≥ 18 years, fluent in the Dutch or English language, between 16 and 26 weeks pregnant at baseline, and high levels of FOC as indicated by a score ≥ 66 on the Wijma-Delivery Expectation Questionnaire (W-DEQ-A) [31]. Exclusion criteria were psychotic episodes, suicidal risk, substance use and dependency, borderline personality disorder, current trauma or traumatic stress disorder, HIV infection, multiple gestations, high risk for premature labour, or participation in other MBPs in the past year.

### Intervention: mindfulness-based childbirth and parenting (MBCP)

The intervention consisted of the face-to-face, group-based MBCP program for expectant parents published as the course book “Mindful Birthing” [32]. MBCP was originally designed to teach life-skills and to promote healthy pregnancy and childbirth to all expectant parents. In our trial, we adapted it for pregnant women with FOC. Adaptations were focused on facilitating participants in every session with skilful responding to anxiety- and fear-related responses in guided meditations and enquiry. The nine weekly sessions, with up to six couples in a group, lasted 3 h, and were delivered by experienced midwives certified in MBCP. Sessions included: mindfulness meditation practice (e.g., body scan, sitting and walking mediations, speaking and listening meditation on fear and happiness, yoga) and enquiry; and teachings about psychobiological processes in childbirth (e.g., physiology of labour pain, dilatation, delivery and postpartum) and in new-borns. Participants were asked to commit to daily meditation practices at home for 30 min. MBCP was free of charge, and the sessions took place at mindfulness centres in Amsterdam and The Hague, The Netherlands. MBCP feasibility and participant’s attendance are presented elsewhere [6].

### Active control condition: enhanced care as usual (ECAU)

ECAU consisted of two individual fear of childbirth consultations of 1.5 h for the expectant couple. Both consultations were spread over a nine-week period (similar to MBCP) and were delivered by trained midwives. ECAU was developed specifically for anxious pregnant women by the research team to reduce FOC by gaining insight into the factors causing and maintaining fear and stress around pregnancy, birth and the postpartum period (the first consultation); and making a coping plan to deal with fears and stressors and discuss psychoeducation about fear (the second consultation). More specifically, the first consultation was based on the Biopsychosocial Model [33], and the second consultation consisted of writing the commonly used Childbirth Plan of the Royal Dutch Organization of Midwives (KNOV) [34]. ECAU was free of charge, and the consultations took place at the couple’s home.

### Measures

#### Time

Measurements of FOC, catastrophic beliefs about labour pain, and mindful awareness were collected at pre-intervention (T1) and post-intervention before childbirth (T2). The childbirth mode including obstetric interventions used during childbirth were collected after birth (T3). Participant characteristics were collected at T1.

#### Pathways of action

##### Emotion pathway: fear of childbirth

The emotion pathway was operationalized as FOC and assessed with the 33-item W-DEQ-A [32]. The questionnaire operationalizes emotions around childbirth (e.g., ‘How do you expect you will feel during delivery; ‘lonely, strong, confident, scared, happy, proud’) as covering general fear, negative appraisal, loneliness, lack of self-efficacy, lack of positive anticipation, and concerns about the child (range 0–165). Higher scores indicate more FOC: *high* (W-DEQ-A ≥ 66); *severe* (W-DEQ-A ≥ 85); and *phobic* FOC (W-DEQ-A ≥ 100) [35]. The W-DEQ-A showed good reliability in an average sample of pregnant women at 16–26 weeks pregnancy (α =0.94) [6]. Cronbach’s α at T1 and T2 in the present study was 0.95.

##### Cognition pathway: catastrophic beliefs

The cognition pathway was operationalized as catastrophic beliefs about labour pain, and it was assessed by the 12-item Catastrophizing Labour Pain (CLP; range 0–60). This subscale is derived from the Labour Pain Cognitions and Coping List (LPCCL) [18]. A higher score on the CLP represents more catastrophizing of labour pain (e.g., “The pain of childbirth will be overpowering”). In the aforementioned study, the CLP showed good reliability in an average sample of pregnant women (30–34 weeks pregnant) with a Cronbach’s α of 0.84. Cronbach’s α in the present study at T1 was 0.88 and at T2 was 0.92.

##### Attention pathway: mindful awareness

The attention pathway in our model was operationalized as mindful awareness. Mindful awareness was assessed with the Dutch version of the 24-item Five Facet Mindfulness Questionnaire (FFMQ; range 24–120) [36]. The FFMQ consists of five subscales: *Observing* (e.g., “When I’m walking, I deliberately notice the sensations of my body moving”); *Describing* (e.g., “I can easily put my beliefs, opinions, and expectations into words”); *Acting with awareness* (e.g., “When I take a shower or bath, I stay alert to the sensations of water on my body”); *Nonjudging of inner experience* (e.g. “I tell myself that I shouldn’t be thinking the way I’m thinking”); and *Nonreactivity to inner experience* (e.g., “I watch my feelings without getting lost in them”). Higher scores indicate greater mindful awareness. Cronbach’s α in the present study at T1 was 0.73 and at T2 was 0.79.

#### Intervention outcome: gradient of childbirth mode

Gradient of childbirth mode was operationalized into an ordinal scale consisting of five categories, with higher scores indicating childbirth with more advanced obstetric interventions: 0 = natural childbirth as birth without any obstetric interventions; 1 = spontaneous childbirth with some obstetric intervention (e.g., augmentation with oxytocin or assisted delivery) not including EA; 2 = spontaneous childbirth with EA; 3 = childbirth with obstetric indication for CS made during childbirth; and 4 = childbirth by sCS.

#### Attendance and practice

The minutes of meditation practice at home were registered by the participants. This data and the presence of participants at MBCP sessions were collected at each of the nine weekly intervention sessions by a MBCP trainer.

### Statistical analysis

The primary analysis was performed using the completers data. The allocation process was concealed from the independent outcome assessor. To test our hypotheses of the three pathways of action in the theoretical model of avoiding versus approaching the challenges of childbirth [8], we ran (1) a parallel, multiple mediation model with our hypothesized mediators and (2) single mediation models to further delineate indirect effects. Each variable was transformed to account for the difference between T1 and T2 (i.e., the change scores (Δ), T2-T1). Our independent variable was dichotomous (i.e., ECAU denoted with 0 and MBCP as 1). According to Hayes (2018), utilizing an ordinal variable as a continuous variable (as we did with our outcome variable) in a statistical mediation model is acceptable [37]. No additional covariates were added to the models since randomization of condition assignment was successful [6].

We conducted mediation analyses using the SPSSv25 PROCESSv3.3 macro [38] to test the hypothesized mediators’ effects of the type of intervention (i.e., MBCP or ECAU) on the gradient of childbirth mode. One model with the following mediators was run: (Δ) W-DEQ-A for FOC; (Δ) CLP for catastrophic beliefs about labour pain; and (Δ) FFMQ for mindful awareness. If a significant indirect effect was found, the effect size for each mediator was estimated using the bootstrapping procedure recommended by Hayes (2018). It accounts for a nonparametric distribution and retains power in the model. We tested whether the specific indirect effect was significantly different from zero by constructing 95% confidence intervals using 10.000 bootstrap samples. If zero is contained in the interval, then the indirect effect is non-significant, suggesting the data do not support the proposed indirect effect. Coefficients, standard errors, and *p*-values were generated [see Additional file 1]. Their corresponding bootstrap confidence intervals were calculated and are documented in Table 3. Note that coefficients are unstandardized, but the bootstrap confidence intervals are standardized [37]. Additionally, indirect paths are partially standardized, which signify the number of standard deviations by which the gradient of childbirth mode is expected to increase/decrease per a change in mediator of size unstandardized coefficient (*a*) [39]. In addition, to assess the relationship between outcome measure and number of completed MBCP sessions and quantity of meditation practice at home per week, Spearman’s rank-order correlations were calculated.

## Results

### Participants’ characteristics

There were no significant pre-intervention differences between participants at T1 in each condition for demographic predictors and outcome measures [6]. Of the 141 participants, two medical files reporting childbirth outcomes were missing (MBCP, *n* = 1; ECAU, *n* = 1). The final sample used for mediation analysis in this study consisted of 139/141 (99.3%) pregnant women with high FOC. Further, we found that the pre- and post-interventions pathways’ measurements were filled in by 113 (81.3%) participants for W-DEQ-A, 109 (78.4%) participants for the CLP, and 111 (79.9%) participants for the FFMQ. There were no statistically significant differences between the participants who did or did not have missing outcome data in the pre-intervention participant characteristics (i.e., group assignment, parity, age and FOC) per pathway.

The sample consisted of 62.6% (*n* = 87) nulliparous and 37.4% *(n* = 52) multiparous pregnant women with an average age of 33 years (*M* = 32.97, *SD* = 3.89). About 75.5% (*n* = 105) of participants reported psychological/psychiatric problems in the past, 25.9% (*n* = 36) used medication for psychological problems more than 1 year, and 19.4% (*n* = 27) were currently in care. The entire sample was characterized with severe FOC (W-DEQ-A, *M* = 93.38, *SD* = 17.90) at the screening, per W-DEQ-A guidelines with a score > 85 indicating severe FOC [31]. Descriptives of each pathway of action measurement are presented in Table 1.

**Table 1:** Pathways of Action: Descriptive Statistics of Variables by Total Sample and Condition

		Intervention Condition	
	Total (*N* = 139) *M (SD)*	MBCP (*n* = 74) *M (SD)*	ECAU (*n* = 65) *M (SD)*
ΔEmotion - FOC (W-DEQ-A)	− 23.04 (21.70) (*n* = 113)	−28.46 (21.35) (*n* = 57)	− 17.54 (20.82) (*n* = 56)
ΔCognition - Labour Pain (CLP)	−9.48 (10.73) (*n* = 109)	−12.89 (10.57) (*n* = 56)	− 5.87 (9.76) (*n* = 53)
ΔAttention - Mindful Awareness (FFMQ)	2.41 (11.30) (*n* = 111)	6.84 (9.82) (*n* = 56)	−2.11 (10.99) (*n* = 55)

*Note.* Δ: difference in post-assessment - pre-assessment, *CLP* Catastrophizing Labour Pain, *ECAU* Enhanced Care As Usual (control group), *FFMQ* Five Facet Mindfulness Questionnaire, *FOC* Fear of Childbirth, *M* mean, *MBCP* Mindful-Based Childbirth and Parenting, *SD* standard deviation, *W-DEQ-A* Wijma Delivery Expectancy Questionnaire

The study’s participants had the following gradient of childbirth mode: 35% (*n* = 49) natural childbirth as spontaneous birth without any obstetric interventions; 6.5% (*n* = 9) spontaneous childbirth with some obstetric intervention (e.g., augmentation with oxytocin or assisted delivery) not including EA; 33.8% (*n* = 47) spontaneous childbirth with EA; 18% (*n* = 25) childbirth with obstetric indication for CS made during childbirth; and 6.5% (*n* = 9) childbirth by sCS. All groups were mutually exclusive.

Further, the average number of completed MBCP sessions was almost 7 out of 9 (*n* = 73, *M* = 6.90; *SD* = 2.83). The average number of minutes per week spent on meditation practice at home within the MBCP program was 85 (*n* = 73, *M* = 85.05, *SD* = 58.96).

### Mediation model

After listwise deletion of participants with missing values, our sample was 109. Descriptives of this sample across pathways of action measures by condition can be found in Table 2.

**Table 2:** Pathways of Action: Descriptive Statistics of Variables by Mediation Sample and Condition

		Intervention Condition	
	Total (*N* = 109) *M* (*SD*)	MBCP (*n* = 56) *M* (*SD*)	ECAU (*n* = 53) *M* (*SD*)
ΔEmotion – FOC (W-DEQ-A)	−22.46 (21.20)	− 27.80 (20.96)	− 16.81 (20.15)
ΔCognition - Labour Pain (CLP)	−9.48 (10.73)	−12.89 (10.57)	−5.87 (9.76)
ΔAttention - Mindful Awareness (FFMQ)	2.29 (11.23)	6.84 (9.82)	−2.53 (10.67)

*Note.* Δ: difference in post-assessment - pre-assessment, *CLP* Catastrophizing Labour Pain, *ECAU* Enhanced Care As Usual (control group), *FFMQ* Five Facet Mindfulness Questionnaire, *FOC* Fear of Childbirth, *M* mean, *MBCP* Mindful-Based Childbirth and Parenting, *SD* standard deviation, *W-DEQ-A* Wijma Delivery Expectancy Questionnaire

**Fig. 1:**
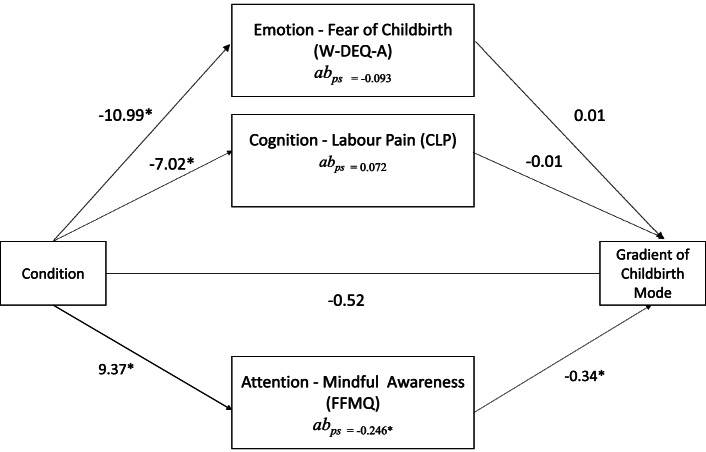
Parallel Mediation Results of Pathways of Action. Note: *n* = 109. Unstandardized regression coefficients (b) for the relationship of intervention condition on the gradient of childbirth mode as mediated by FOC, catastrophizing beliefs of labour pain, and mindful awareness. Indirect paths are partially standardized (abps), and pathways are change scores between the difference in post-assessment and pre-assessment. *p < 0.05

Results indicated that the direct effect in the full model including mediators was non-significant (*p* = 0.0529; 95% CI [− 1.036, 0.007]). The indirect effect (ab_ps_ = − 0.246) of mindful awareness was significant (95% CI [− 0.428, − 0.093]). These results indicate a full mediation with the mediators, however, note that the direct effect is approaching significance. Moreover, approximately 21% of the variance (R^2^ = 0.21, *F*[4104] = 6.896, *p* = 0.0001) in the gradient of childbirth mode was accounted for by these mediators. This is indicated by the indirect effect (ab_ps_ = − 0.267 units; 95% CI [− 0.480, − 0.066]). When examining each of the three indirect effects, we found that catastrophic beliefs of labour pain and FOC were non-significant mediators (see Fig. 1). Note that mindful awareness accounts for a large portion of the effect in the total indirect effect. See Table 3 for total, direct and indirect effects.

**Table 3:** Effects of Intervention Condition (X) on Gradient of Childbirth Mode (Y) with Parallel Mediation

			95% Confidence Interval^a^	
	Partial Std Effect	SE	Lower	Upper
Model 1				
Total effect of X on Y	− 0.652***	0.243	−1.354	−0.390
Direct effect of X on Y	−0.385^b^	0.263	−1.036	0.007
			Bootstrap 10.000 Times 95% Confidence Interval, Standardized	
Cond – Total Indirect Effect – Y	−0.267	0.105	**−0.480**	**− 0.066**
Cond – ΔEmotion: FOC (W-DEQ-A) – Y	−0.093	0.069	−0.246	0.026
Cond – ΔCognition: Labour Pain (CLP) – Y	0.072	0.087	−0.096	0.257
Cond – ΔAttention: Mindful Awareness (FFMQ) – Y	−0.246	0.084	**−0.428**	**−0.093**

*Note. n* = 109. Δ: difference in post-assessment - pre-assessment, *CLP* Catastrophizing Labour Pain, *FFMQ* Five Facet Mindfulness Questionnaire, *W-DEQ-A* Wijma Delivery Expectations Questionnaire

^a^Bootstrap confidence intervals are fully standardized (Hayes, 2018). Bolded bootstrap CI indicate significant indirect paths. If zero is contained in the interval, then the indirect effect is non-significant, suggesting the data do not support the proposed indirect effect. Indirect, direct, and total effects are partially standardized (std)

^b^The direct effect is partially standardized here whereas in Fig. 1 it is unstandardized

*p ≤ 0.05, **p < 0.01, ***p < 0.001

### Mindful awareness as a mediator

As our results show, mindful awareness is the only significant mediator, which accounts for approximately 18% (R^2^ = 0.18, *F*[1107] = 22.77, *p* < 0.0001) of the variance in the gradient of childbirth mode outcome. To further understand the specifics behind the mechanism of change in mindful awareness, we conducted post-hoc, exploratory analyses with the entire sample who completed the mindful awareness measure (FFMQ; *n* = 111). We ran single mediation analyses with each of the five subscales of mindful awareness. For coefficients, standard errors, *p-*values and R^2^ [see Additional file 2]. *Nonreactivity to inner experience* significantly mediated the effect of intervention condition on the gradient of childbirth mode (*ab*_*ps*_ = − 0.110; 95% CI = [− 0.247, − 0.006]). The other four subscales showed non-significant indirect effects. To understand the variance amongst the five subscales, we ran a parallel model where all five subscales were included. For coefficients, standard errors, *p-*values and R^2^, [see Additional file 3]. This model accounts for approximately 23% (R^2^ = 0.23, *F*[6104] = 5.17, *p* = 0.0001) of the variance in the gradient of childbirth mode outcome. All five subscales had a non-significant indirect effect though *Nonreactivity to inner experience* was nearly significant (*ab*_*ps*_ = − 0.091, 95% CI = [− 0.2162, − 0.0004]), and it accounted for the largest effect in the model as compared to the other four mindful awareness subscales. See Table 4 for total, direct and indirect effects.

**Table 4:** Intervention Condition (X) on Gradient of Childbirth Mode (Y) through FFMQ Subscale Mediators - Single and Parallel Mediation

	Partial Std Effect	SE	95% Confidence Interval	
			Lower	Upper
Single Mediation				
Total effect of X on Y	−0.659***	0.239	−1.347	−0.401
Direct effect of X on Y	−0.549**	0.234	−1.192	−0.266
Indirect: Cond – ΔNOR – Y	−0.110	0.061	**−0.247**	**−0.006**
Total effect of X on Y	−0.659***	0.239	−1.347	−0.401
Direct effect of X on Y	−0.596**	0.244	−1.274	−0.308
Indirect: Cond – ΔNOJ – Y	−0.063	0.045	−0.158	0.018
Total effect of X on Y	−0.657***	0.239	−1.347	−0.401
Direct effect of X on Y	−0.652***	0.251	−1.362	−0.368
Indirect: Cond – ΔACT – Y	−0.007	0.064	−0.127	0.133
Total effect of X on Y	−0.659***	0.239	−1.347	−0.401
Direct effect of X on Y	−0.577**	0.251	−1.264	−0.269
Indirect: Cond – ΔDES – Y	−0.081	0.060	−0.207	0.030
Total effect of X on Y	−0.659***	0.239	−1.347	−0.401
Direct effect of X on Y	−0.598**	0.236	−1.260	−0.326
Indirect: Cond – ΔOBS – Y	−0.061	0.050	−0.173	0.021
Parallel Mediation				
Total effect of X on Y	−0.657***	0.239	−1.347	−0.401
Direct effect of X on Y	−0.474*	0.250	−1.125	−0.132
Indirect				
Cond – Total – Y	−0.185	0.104	−0.380	0.029
Cond – ΔNOR – Y	−0.091	0.065	**−0.216**	**< 0.001**
Cond – ΔNOJ – Y	−0.065	0.049	−0.170	0.023
Cond – ΔACT – Y	0.019	0.068	−0.102	0.177
Cond – ΔDES – Y	−0.001	0.063	−0.118	0.139
Cond – ΔOBS – Y	−0.047	0.042	−0.146	0.017

*Note. n = 111.* Δ: difference in post-assessment - pre-assessment, *ACT* Acting with awareness, *DES* Describing, *FFMQ* Five Facet Mindfulness Questionnaire, *NOJ* Nonjudging of inner experience, *NOR* Nonreactivity to inner experience, OBS Observing

^a^Indirect, direct, and total effects are partially standardized (std)

^b^Indirect effects reflect a 10.000 bootstrap sampling with confidence interval (CI) of 95%, and CIs are fully standardized (Hayes, 2018). Bolded bootstrap CI indicate significant indirect path. If zero is absent from the interval, then the indirect effect is significant, suggesting the data support the proposed indirect effect

*p ≤ 0.05, **p < 0.01, ***p < 0.001

### Amount of mindfulness practice

There was no association between the gradient of childbirth mode and number of attended MBCP sessions (r_s_ = 0.020, *p* = 0.08, *n* = 73). However, there was a significant moderate, positive correlation between total minutes per week meditated at home and the gradient of childbirth mode (r_s_ = 0.39, *p* = 0.001, *n* = 72).

## Discussion

### Main findings

The aim of this study was to clarify *how* participation in an MBCP program could lead to a natural childbirth (the lowest gradient of childbirth mode) in pregnant women with high FOC. For this purpose, we examined three pathways of action that would operate with adaptation to childbirth through natural childbirth: emotion (FOC), cognition (catastrophic beliefs about labour pain) and attention (mindful awareness). Our results showed that MBCP increases natural childbirths through an increase in mindful awareness, and in particular, nonreactivity to inner experiences. Neither a decrease in FOC or catastrophic beliefs about labour pain were found to be mechanisms of change. In addition, natural childbirth was positively associated with minutes of meditation practice. The more one meditated, the more one was inclined towards a natural childbirth and vice versa. No relation was found between the attendance to MBCP sessions and natural childbirth.

### Interpretation

These results are novel and interesting in the field of psychosomatic care for pregnant women, as they lead us to draw several conclusions. The main conclusion is that the change in the quality of attention (increased mindful awareness) in pregnant women with high FOC, and not the change in their negative cognitive-emotional states (decreased FOC and catastrophic beliefs), is the important mechanism of change. This mechanism leads to being able to approach the challenges of childbirth and adapt to them, rather than avoid them. Extant research has focused on the contribution of the cognitive-emotional state of pregnant women in relation to the use and requests for obstetric interventions during childbirth [11–20]. However, our results show that the quality of attention (increased mindful awareness) seems to be superior in approaching and adapting (natural childbirth) the challenges of childbirth in pregnant women with high FOC.

Research on mindful awareness and behaviour regulation in populations with emotion dysregulation showed that approaching and observing their intense emotions may improve their ability to tolerate negative emotional states and cope with them effectively [21, 26, 27]. It is likely that pregnant women who attended a MBCP training developed the ability to approach and to notice their FOC and catastrophic beliefs, and thus, were able to tolerate them during childbirth. Mindfulness practice in a MBCP training encourages awareness of all cognitive, emotional and bodily states related to childbirth while being non-judgmental and nonreactive, and allows the challenges belonging to childbirth to exist. In other words, maintaining a mindful awareness towards fearful emotions and catastrophic beliefs about childbirth, without judgment and without reactivity, may help to regulate behaviour from being reactive and avoiding the challenges of childbirth to shift to being more responsive and approaching, and adapting to the childbirth process.

Our study shows that being non-reactive to the inner experience (a facet of mindful awareness) caused more adaptation to childbirth (more natural childbirths). The adaptation to childbirth through nonreactivity to inner experience may be explained by less stress. This suggestion is supported by Lönnberg and colleagues (2020), who found that an increase in nonreactivity to inner experience was significantly correlated with a reduction in perceived stress in pregnant women in MBCP as compared to Lamaze childbirth classes [5]. It could be concluded that after MBCP, pregnant women in our study experienced childbirth as less stressful or threatening and allowed themselves to undergo and to adapt more to the processes of natural childbirth than pregnant women in the control group. In light of these findings, it would be relevant to study the biological effects of mindful awareness in pregnant women with high FOC by measuring perceived stress symptoms and levels of the maternal stress hormone cortisol. High levels of maternal cortisol limit the DNA expression in children born from mothers with high FOC [40]. By reducing the maternal cortisol level, the health potential of a new-born could be improved [40].

Further, our results demonstrate that natural childbirth was positively related to more extensive home meditation practice. This finding is supported by a study of home meditation practice for depression relapse-prevention, which showed that more minutes spent on mindfulness meditation was associated with a lower hazard of relapse to depression [38]. However, Lönnberg’s study showed no relationship between minutes spent on mindfulness meditation and change in perceived stress in pregnant women (2020). Based on our results, it could be concluded that more extensive home meditation practice is associated with natural childbirth and vice versa thus indicating better adaptation to the challenges of childbirth through more extensive mindful awareness. Noteworthy, the actual number of MBCP sessions attended in our study was not related to the gradient of childbirth mode. However, most MBCP participants attended at least seven out of nine sessions. It could be interpreted that apparently practicing on a regular, daily basis seems to be extra important for the effect than being only present during MBCP sessions.

Importantly, in accordance with psychological theories such as cognitive theory [9] and experiential avoidance theory [41], it is not the actual negative emotions, negative beliefs or unpleasant sensations, but how one responds to them (approaching versus avoiding), that is linked to a wide range of mental health issues. In view of these theories, avoiding experiences that are appraised as threatening may reduce distress in the short term in pregnant women with high FOC, but it may maintain and even reinforce fear and anxiety in future pregnancies and childbirths. So far, only cognitive behaviour therapy (CBT) [9] delivered by psychotherapists has demonstrated improved adaptation to childbirth with an increase in natural births and a decrease in the use of sCS in pregnant women with high FOC [42]. CBT is a clinical and relatively expensive intervention when compared to MBCP, which is a non-clinical intervention that can be delivered by other professionals caring for pregnant women. Given that high FOC is quite prevalent (25%) [43] and childbirth medicalisation is growing worldwide (WHO, 2019) [44], effective care that addresses these issues is of paramount importance, both for health-economic (e.g., cost of medical care) and health-psychological (e.g., emotional burden of high FOC and associated requested obstetric interventions) reasons. On the other hand, pregnant women with high FOC who underwent natural childbirth possibly without adequate preparation, experienced childbirth as traumatic, and even developed post-traumatic stress disorder, post-partum depression and persistent pain after childbirth [45]. Noteworthy is that several factors complicate intrapartum care for pregnant women with high FOC: the impact of FOC on additional mental health issues; the advantages and disadvantages of EA and CS; and pregnant women’s right to demand the care they prefer. To tackle this complexity in the care for pregnant women with high FOC, interventions should be offered that: support pregnant women in dealing with the challenges of childbirth; and provide tailored information and support about FOC, as well as about the advantages, and disadvantages of obstetric interventions in childbirth.

Given that natural childbirth optimally supports production of oxytocin in the mother’s brain and positively influences maternal physiology and behavior during childbirth, and motherhood [46], natural childbirth with an optimal oxytocin level could be seen as an important intervention in treatment of internalizing and externalizing problems in mothers and their children [47]. Having a spontaneous childbirth without obstetric interventions may reduce the chance for postpartum depression and/or development of posttraumatic stress disorder [45]. Positive body and mind experiences may empower motherhood and improve the mother-child relationship especially in the context of fear [47].

To further this line of work, future studies should be performed in large and heterogeneous populations of pregnant women and in a multicentered design. Further studies exploring the active ingredients of the MBCP program and implementation factors related to the delivery of MBCP such as the quality of trainer, as well as time spent on the meditation practice and attendance to the sessions in adaptation to childbirth in pregnant women with high FOC, could be of interest. Importantly, the effect of MBCP on the perceived stress reduction and the reduction in maternal cortisol level require more attention in future research, given the knowledge about the extended harmful effects for the unborn child in pregnant women with high FOC [48, 49]. However, the most pressing recommendation is a replication of this study to confirm our findings and reinforce the implications.

### Strengths and limitations

To the best of our knowledge, this study is the first to explore mechanisms of change operating through three psychological pathways of action, for adaptation to childbirth in pregnant women with high FOC, by comparing the effects of MBCP with ECAU. Our results contribute to a richer understanding of underlying psychological mechanisms through which adaptation to a (natural) childbirth may operate. Second, the study design and statistical analyses generally meet the standards of mechanisms of change study guidelines, as defined in a systematic review on RCTs examining potential mechanisms of change in MBPs [1]. The mediation analyses were conducted in an RCT with an active control group and the outcome assessor was blinded to the allocation process. The mechanisms of change hypothesized in this study were drawn from a psychological model of the potential pathways of action already introduced in a protocol study, which presented substantiation for the model and the expected changes [30]. Importantly, in our mediation analyses, change in the mediator preceded the change in the outcome in time (i.e., temporal precedence) [37]; with this, an alternative explanation for the change in mindful awareness and change in the gradient of advanced obstetric interventions used during childbirth were eliminated. Consequently, this study provides sufficient evidence to conclude that increased mindful awareness is the causal mechanism that explains more adaptation to natural childbirth and a decrease in obstetric interventions during childbirth, as observed in the MBCP participants compared to ECAU participants.

This study had several limitations. Our sample mainly consisted of Caucasian, highly educated pregnant women, which limits generalizability of the results to more diverse populations. Although our analyses indicated that there was no selective drop-out or missing values, selective attrition could be a limitation, and this may also have limited the power to detect effects. More studies with larger and more diverse populations are warranted.

## Conclusions

Cultivation of greater mindful awareness, and more specifically nonreactivity to inner experience, in pregnant women with high FOC during the nine-week MBCP program appears to be a mechanism of change leading to natural childbirths and less use of obstetric interventions, such as sCS. In addition, meditating more often appears to be related to a higher degree of natural childbirth, and vice versa, but higher attendance to MBCP sessions was not. Whether these findings have wider application deserves further study and attention from healthcare providers and policymakers.

## Supplementary Information

**Additional file 1.** Regression Coefficients, Standard Errors, and Summary Information for Parallel Mediation Models of Condition on Gradient of Childbirth Mode.

**Additional file 2.** Regression Coefficients, Standard Errors, and Summary Information for five FFMQ Subscales Mediating the Condition on Gradient of Childbirth Mode.

**Additional file 3.** Regression Coefficients, Standard Errors, and Summary Information for the 5 FFMQ Subscales Parallel Mediation of Condition on Gradient of Childbirth Mode.

## Data Availability

The datasets analysed during current study are available from the corresponding author upon reasonable request.

## Abbreviations

EA: Epidural analgesia
ECAU: Enhanced care as usual
CLP: Catastrophizing Labour Pain
CT: Cognitive Therapy
CBT: Cognitive Behavioural Therapy
CS: Cesarean section
FFMQ: Five Facets Mindfulness Questionnaire
FOC: Fear of childbirth
KNOV: Koninklijke Nederlands Organisatie van Verloskundigen
LPCCL: Labour Pain Coping Cognitions List
MBCP: Mindfulness-Based Childbirth and Parenting
MBCT: Mindfulness-Based Cognitive Therapy
MBPs: Mindfulness-Based Programs
MBSR: Mindfulness-Based Stress Reduction
RCT: Randomized controlled trial
sCS: Self-requested cesarean section
PTSD: Posttraumatic stress disorder
VAS: Visual Analogue Scale
W-DEQ-A: Wijma Delivery Expectation Questionnaire
