# Supplementary material for: Mindful awareness as a mechanism of change for natural childbirth in pregnant women with high fear of childbirth: a randomised controlled trial
Source: BMC Pregnancy Childbirth. 2022 Jan 19;22:47. doi: 10.1186/s12884-022-04380-0 (PMC8767678; doi:10.1186/s12884-022-04380-0)
Supplement: Supplementary file 1 — Additional file 1. Regression Coefficients, Standard Errors, and Summary Information for Parallel Mediation Models of Condition on Gradient of Childbirth Mode. [file 12884_2022_4380_MOESM1_ESM.docx]

| **Additional file 1** |  |  |  |  |  |  |  |  |  |  |  |  |  |  |  |  |  |
| --- | --- | --- | --- | --- | --- | --- | --- | --- | --- | --- | --- | --- | --- | --- | --- | --- | --- |
| Regression Coefficients, Standard Errors, and Summary Information for Parallel Mediation Models of Condition on Gradient of Childbirth Mode | | | | | | | | | | | | | | | | |  |
|  |  |  |  |  |  | Consequent |  |  | | |  |  |  |  |  |  |  |
|  |  |  |  |  |  |  |  |  |  |  |  |  |  |  |  |  |  |
|  |  | *M*_1_ (∆FFMQ) | |  |  | *M*_2_ (∆W-DEQ-A) | | |  | *M*_3_ (∆CLP) | |  |  | *Y* (Gradient of Childbirth Mode) | | |  |
| Antecedent |  | Coeff. | *SE* | *p* |  | Coeff. | *SE* | *p* |  | Coeff. | *SE* | *p* |  | Coeff. | *SE* | *p* |  |
| *X* (COND) | *a*_1_ | 9.367 | 1.963 | < 0.001 | *a*_2_ | -10.992 | 3.942 | 0.006 | *a*_3_ | -7.025 | 1.951 | < 0.001 | *c*_1_ | -0.515 | 0.263 | 0.053 |  |
| M1 (∆FFMQ) |  | --- | --- | --- |  | --- | --- | --- |  | --- | --- | --- | *b*_1_ | -0.035 | 0.012 | 0.004 |  |
| M2 (∆W-DEQ-A) |  | --- | --- | --- |  | --- | --- | --- |  | --- | --- | --- | *b*_2_ | 0.011 | 0.007 | 0.108 |  |
| M3 (∆CLP) |  | --- | --- | --- |  | --- | --- | --- |  | --- | --- | --- | *b*_3_ | -0.014 | 0.014 | 0.328 |  |
| Constant | *i*_M1_ | -2.529 | 1.407 | 0.075 | *i*_M2_ | -16.811 | 2.825 | < 0.001 | *i*_M3_ | -5.868 | 1.399 | < 0.001 | *i*_Y_ | 1.964 | 0.200 | < 0.001 |  |
|  |  | *R*_2_ = 0.175 |  |  |  | *R*_2_ = 0.068 |  |  |  | *R*_2_ = 0.108 |  |  |  | *R*_2_ = 0.210 |  |  |  |
|  |  | *F*(1,107) = 22.765, |  |  |  | *F*(1,107) = 7.776, |  |  |  | *F*(1,107) = 12.961, |  |  |  | *F*(4,104) = 12.961, |  |  |  |
|  |  | p < 0.001 |  |  |  | *p* = 0.006 |  |  |  | *p* < 0.001 |  |  |  | *p* < 0.001 |  |  |  |
|  |  |  |  |  |  |  |  |  |  |  |  |  |  |  |  |  |  |
| *Note. n=109*.∆: difference in post-assessment - pre-assessment; *a* = path of X variable to mediator; *b* = path of mediator to outcome *Y*; c1= direct effect path; CLP = Catastrophizing Labour Pain; FFMQ = Five Facet Mindfulness Questionnaire; W-DEQ-A = Wijma Delivery Expectations Questionnaire.  Coefficients are unstandardized. | | | | | | | | | | | | | | | | |  |
|  |  |  |  |  |  |  |  |  |  |  |  |  |  |  |  |  |  |
