# Supplementary material for: Mindful awareness as a mechanism of change for natural childbirth in pregnant women with high fear of childbirth: a randomised controlled trial
Source: BMC Pregnancy Childbirth. 2022 Jan 19;22:47. doi: 10.1186/s12884-022-04380-0 (PMC8767678; doi:10.1186/s12884-022-04380-0)
Supplement: Supplementary file 2 — Additional file 2. Regression Coefficients, Standard Errors, and Summary Information for five FFMQ Subscales Mediating the Condition on Gradient of Childbirth Mode. [file 12884_2022_4380_MOESM2_ESM.docx]

| **Additional file 2**  *Regression Coefficients, Standard Errors, and Summary Information for five FFMQ Subscales Mediating the Condition on Gradient of Childbirth Mode* | | | | |
| --- | --- | --- | --- | --- |
| Effect |  | Gradient of Childbirth Mode | | |
|  |  | *b* | *SE*(*b*) | 95% CI |
| ΔNOR |  |  |  |  |
| a |  | 1.677* | 0.814 | [-1.074, 1.219] |
| b |  | -0.087** | 0.027 | [-0.140, -0.033] |
| c_1_ |  | -0.729** | 0.234 | [-1.192, -0.266] |
| R^2^ |  | 0.187  12.423*** | | |
| *F*(2, 108) |  |  |  |  |
| ΔNOJ |  |  |  |  |
| a |  | 2.083* | 0.868 | [0.362, 3.803] |
| b |  | -0.040 | 0.026 | [-0.092, 0.012] |
| c_1_ |  | -0.791** | 0.244 | [-1.274, -0.308] |
| R^2^ |  | 0.128  7.929*** | | |
| *F*(2, 108) |  |  |  |  |
| ΔDES |  |  |  |  |
| a |  | 2.030*** | 0.583 | [0.877, 3.185] |
| b |  | -0.053 | 0.039 | [-0.131, 0.025] |
| c_1_ |  | -0.766** | 0.251 | [-1.264, -0.269] |
| R^2^ |  | 0.124  7.665*** | | |
| *F*(2, 108) |  |  |  |  |
| ΔOBS |  |  |  |  |
| a |  | 0.842 | 0.575 | [-0.299, 1.982] |
| b |  | -0.096* | 0.039 | [-0.173, -0.019] |
| c_1_ |  | -0.793** | 0.236 | [-1.260, -0.326] |
| R^2^ |  | 0.157  10.067*** | | |
| *F*(2, 108) |  |  |  |  |
| ΔACT |  |  |  |  |
| a |  | 2.317** | 0.729 | [0.871, 3.762] |
| b |  | -0.004 | 0.032 | [-0.067, 0.058] |
| c_1_ |  | -0.865*** | 0.251 | [-1.362, -0.368] |
| R^2^ |  | 0.110  6.643** | | |
| *F*(2, 108) |  |  |  |  |
| *Note. n=111.* Δ: difference in post-assessment - pre-assessment; ACT = Acting with awareness; DES = Describing; FFMQ = Five Facet Mindfulness Questionnaire; NOJ = Nonjudging of inner experience; NOR = Nonreactivity to inner experience; OBS = Observing. ^a^Coefficients are unstandardized (b).  **p* ≤ .05, ***p* < .01, ****p* < .001. | | | | |
