# Supplementary material for: Mindful awareness as a mechanism of change for natural childbirth in pregnant women with high fear of childbirth: a randomised controlled trial
Source: BMC Pregnancy Childbirth. 2022 Jan 19;22:47. doi: 10.1186/s12884-022-04380-0 (PMC8767678; doi:10.1186/s12884-022-04380-0)
Supplement: Supplementary file 3 — Additional file 3. Regression Coefficients, Standard Errors, and Summary Information for the 5 FFMQ Subscales Parallel Mediation of Condition on Gradient of Childbirth Mode. [file 12884_2022_4380_MOESM3_ESM.docx]

| **Additional file 3** | |  |  |  |  |  |  |  |  |  |  |  |  |  |  |  |  |  |  |  |  |  |  |  |
| --- | --- | --- | --- | --- | --- | --- | --- | --- | --- | --- | --- | --- | --- | --- | --- | --- | --- | --- | --- | --- | --- | --- | --- | --- |
| Regression Coefficients, Standard Errors, and Summary Information for the 5 FFMQ Subscales Parallel Mediation of Condition on Gradient of Childbirth Mode | | | | | | | | | | | | | | | | | | | | | | |  |  |
|  |  |  | |  |  |  |  |  |  |  | Consequent | |  |  |  |  |  |  |  |  |  |  |  |  |
|  |  | *M*_1_ (∆NOR) | |  |  | *M*_2_ (∆NOJ) | |  |  | *M*_3_ (∆DES) | |  |  | *M*_4_ (∆OBS) | |  |  | *M*_5_ (∆ACT) | |  |  | *Y* (Gradient of | |  |
|  |  |  |  |  |  |  |  |  |  |  |  |  |  |  |  |  |  |  |  |  |  | Childbirth Mode) | | |
|  |  | Coeff. | *SE* | *p* |  | Coeff. | *SE* | *p* |  | Coeff. | *SE* | *p* |  | Coeff. | *SE* | *p* |  | Coeff | *SE* | *p* |  | Coeff. | *SE* | *p* |
| Antecedent |  |  |  |  |  |  |  |  |  |  |  |  |  |  |  |  |  |  |  |  |  |  |  |  |
| *X* (COND) | *a_1_* | 1.677 | 0.814 | 0.042 | *a*_2_ | 2.083 | 0.868 | 0.012 | *a*_3_ | 2.030 | 0.582 | < 0.001 | *a*_4_ | 0.842 | 0.575 | 0.147 | *a*_5_ | 2.317 | 0.729 | 0.002 | *c*_1_ | -0.629 | 0.250 | 0.014 |
| *M*_1_ (∆NOR) |  | --- | --- | --- |  | --- | --- | --- |  | --- | --- | --- |  | --- | --- | --- |  | --- | --- | --- | *b*_1_ | -0.072 | 0.029 | 0.016 |
| *M*_2_ (∆NOJ) |  | --- | --- | --- |  | --- | --- | --- |  | --- | --- | --- |  | --- | --- | --- |  | --- | --- | --- | *b*_2_ | -0.042 | 0.026 | 0.119 |
| *M*_3_ (∆DES) |  | --- | --- | --- |  | --- | --- | --- |  | --- | --- | --- |  | --- | --- | --- |  | --- | --- | --- | *b*_3_ | -0.001 | 0.041 | 0.982 |
| *M*_4_ (∆OBS) |  | --- | --- | --- |  | --- | --- | --- |  | --- | --- | --- |  | --- | --- | --- |  | --- | --- | --- | *b*_4_ | -0.074 | 0.039 | 0.063 |
| *M*_5_ (∆ACT) |  | --- | --- | --- |  | --- | --- | --- |  | --- | --- | --- |  | --- | --- | --- |  | --- | --- | --- | *b*_5_ | 0.011 | 0.032 | 0.731 |
| Constant | *i*_M1_ | 0.073 | 0.579 | 0.900 | *i*_M2_ | -0.636 | 0.617 | 0.304 | *i*_M3_ | -0.690 | 0.414 | 0.098 | *i*_M4_ | -0.127 | 0.409 | 0.756 | *i*_Y_ | -0.727 | 0.518 | 0.163 | i_Y_ | 1.922 | 0.165 | <0.001 |
|  |  | *R*_2_ = 0.038 | |  |  | *R*_2_ = 0.050 | |  |  | *R*_2_ = 0.100 | |  |  | *R*_2_ = 0.019 | |  |  | *R*_2_ = 0.085 | |  |  | *R*_2_ = 0.230 | |  |
|  |  | *F*(1,109) = 4.241, | | |  | *F*(1,109) = 5.758, | | |  | *F*(1,109) = 12.147, | | |  | *F*(1,109) = 2.139, | | |  | *F*(1,109) = 10.091, | | |  | *F*(6,104) = 5.168, | | |
|  |  | *p* = 0.042 | |  |  | *p* = 0.019 | |  |  | *p* < 0.001 | |  |  | *p* = 0.147 | |  |  | *p* = 0.002 | |  |  | *p* < 0.001 | |  |
| *Note*. n=111.∆: difference in post-assessment - preassessment; ; *a* = path of X variable to mediator; *b* = path of mediator to outcome Y; *c*^1^= direct effect path; ACT=Acting with awareness; DES = Describing; FFMQ = Five Facet Mindfulness Questionnaire; NOJ = Nonjudging to inner experience; NOR = Nonreactivity to inner experience; OBS = Observing. | | | | | | | | | | | | | | | |  |  |  |  |  |  |  |  |  |
| ^a^Coefficients are unstandardized. | | | |  |  |  |  |  |  |  |  |  |  |  |  |  |  |  |  |  |  |  |  |  |
